# Exploring the diverse applications of sol–gel synthesized CaO:MgAl_2_O_4_ nanocomposite: morphological, photocatalytic, and electrochemical perspectives

**DOI:** 10.1186/s11671-024-04093-7

**Published:** 2024-09-12

**Authors:** H. K. Jahnavi, S. Rajendra Prasad, H. P. Nagaswarupa, Ramachandra Naik, N. Basavaraju, C. R. Ravikumar, Burragoni Sravanthi Goud, Jae Hong Kim

**Affiliations:** 1 Department of Studies in Chemistry, Shivagangothri, Davangere University, Davanagere, 577007 India; 2 Department of Physics, New Horizon College of Engineering, Bangalore, 560103 India; 3 Research Centre, Department of Science, East West Institute of Technology, VTU, Bangalore, 560091 India; 4 School of Chemical Engineering, Yeungnam University, Gyeongsan, 38541 Republic of Korea; 5 Department of Chemistry, School of Sciences, SR University, Warangal, 506371 India

**Keywords:** CaO:MgAl_2_O_4_ nanocomposite, Battery, Sensors, Photocatalytic activity, Plant growth

## Abstract

A nanocomposite of CaO:MgAl_2_O_4_ was synthesized through a straightforward and cost-effective sol–gel method. The investigation of the novel CaO:MgAl_2_O_4_ nanocomposite encompassed an examination of its morphological and structural alterations, as well as an exploration of its photocatalytic activities and electrochemical characteristics. XRD analysis revealed a nanocomposite size of 24.15 nm. The band gap, determined through UV studies, was found to be 3.83 eV, and scanning electron microscopy (SEM) illustrated flake-like morphological changes in the CaO:MgAl_2_O_4_ samples. TEM, HRTEM, and SAED studies of a CaO:MgAl_2_O_4_ nanocomposite would reveal important details about its morphology, crystallography, and nanostructure. Photocatalytic activity was quantified by studying the degradation of Acid Red-88 (AR-88) dye in a deionized solution, achieving a 70% dye degradation under UV irradiation in 120 min. Plant growth examinations were carried out using dye degraded water to test its suitability for agriculture. The electrochemical energy storage and sensing applications of the prepared nanocomposite were examined using CaO:MgAl_2_O_4_ modified carbon paste electrode through cyclic voltammetry (CV) and electrochemical impedance spectroscopy (EIS). In conclusion, the synthesized CaO:MgAl_2_O_4_ nanocomposite demonstrated promising morphological and structural characteristics, efficient photocatalytic activity, and potential applications in electrochemical energy storage, highlighting its versatility for various technological and environmental applications.

**Graphical abstract:**

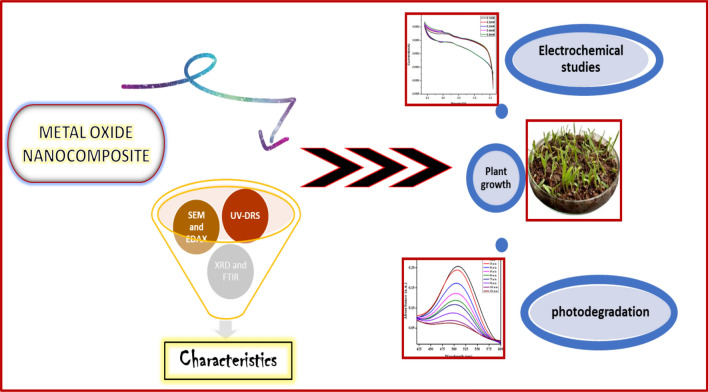

## Introduction

Recent advancements in nanotechnology have led to the development of nanocomposite materials with precise size distribution and monodispersed properties, particularly focusing on metal-oxide nanocomposites with enhanced characteristics [1, 2]. Transition metal oxide nanocomposites have been extensively explored for diverse applications, including supercapacitors, sensors, and photocatalysis [3–7]. Studies suggest that modifying these transition metal oxides with various metal oxide nanoparticles can enhance their effectiveness, introducing new scientific possibilities [8].

Several transition metal oxide nanocomposites, such as the carbon nanotube-ZnO nanocomposite for supercapacitor electrodes [9], the antibacterial activity of CuO–ZnO nanocomposites [10], and the synthesized GO/ZnO nanocomposite for photocatalytic degradation [11], have demonstrated practical applications. While numerous studies have been published on various nanocomposites, including Polyaniline/ZnO for sensing materials [12] and Graphene-ZnO for photovoltaic cells [13], there is currently no published synthesis of a CaO:MgAl_2_O_4_ nanocomposite. CaO nanoparticles, although rarely studied, offer advantages, especially when combined with transparent magnesium aluminate (MgAl_2_O_4_), known for its mechanical and optical qualities and cost-effective production in large quantities [14].

Energy storage and conservation are crucial strategies to manage global energy crises, addressing social and ecological needs by developing modern energy distribution systems [15]. The electrochemical capacitors, known as supercapacitors, offer high power capabilities, outstanding reversibility, and extended life cycles [16]. The electric double layer capacitor (EDLC) technology, utilizing two identical carbon electrodes in aqueous electrolyte for ion adsorption, has been a conventional design [17]. Improving supercapacitor performance involves exploring composites and metal oxide compounds as potential electrode materials. Composite nanomaterials with many functions are extensively employed in several fields such as photocatalysis, sensors, microwave devices, battery anode materials, and solar cells [18].

The photocatalysis approach is a viable and promising method for degrading dyes without generating any secondary pollutants. Moreover, photocatalysis is inexpensive, safe, low energy consumption, and reusable [19]. The effluent from the textile sector contains multiple kinds of hazardous dyes, including methyl orange, acid red, methyl blue, rhodamine etc. Among numerous dyes, AR-88 is often used for diverse applications in the industry and has been discovered to be a stable dye [20]. Due to growing industrialization, which has increased the level of water pollution, environmental contamination is currently a serious problem in both developed and developing countries worldwide [21]. AR-88 is one of the numerous colouring agents that is frequently used in the textile industry for dying as well as in the medical field for staining during surgery and diagnostic testing. Furthermore, these dyes cause severe poisoning, growth-oriented, neurological, reproductive problems and other serious health problems. AR-88 dye degrades when exposed to UV light due to its ability to absorb UV light and suppress the photo-induced e−/h+ recombination state. Due to the existence of various metal oxidation states and the distinct structure of anions attached to these metals as counter anions, scientists have explored the synthesis of bimetallic compounds for electrochemical purposes [21].

In our study, the focus on the CaO:MgAl_2_O_4_ nanocomposite revealed a significant 70% dye degradation in the presence of AR-8 dye, suggesting possibilities for enhanced electrochemical studies and photocatalytic degradation with lower activation energy [22].

Our work involved the synthesis of the CaO:MgAl_2_O_4_ nanocomposite using the sol–gel method. Characterization through XRD, UV-DRS, FT-IR, SEM, and EDXA techniques, along with the investigation of electrochemical performance by employing the nanocomposite as a working electrode, provided valuable insights. The study aimed to explore the photocatalytic activity of the nanocomposite, especially in reducing pollutants in wastewater and assessing the potential for reusing treated wastewater in crop growth.

## Materials and methods

Magnesium nitrate (Mg(NO_3_)_2_·6H_2_O), 99.9%), aluminium nitrate ((Al(NO_3_)_3_·9H_2_O),99.9%), calcium nitrate ((Ca(NO_3_)_2_·4H_2_O),99.9%), sodium hydroxide (NaOH) and urea ((NH_2_CONH_2_),99.9%) as a fuel were bought through Sigma Aldrich. India. Each individual chemicals were utilised without additional purification.

### Synthesis of CaO nanoparticle

Cost-effective CaO nanoparticles (NPs) were produced via a sol–gel method. In this process, stoichiometric amounts of calcium nitrate [Ca(NO_3_)_2_·4H_2_O] and urea [NH_2_CONH_2_] were mixed in a beaker, and a 100 mL of deionized water was added. Urea is a chemical compound utilized as a hydrolysing agent in the synthetic process. Urea has a major effect on the final product nanocomposite’s shape, textural characteristics, and crystal structure. In a typical sol–gel synthesis, urea can be employed as a fuel to generate metal oxides. The mixture was stirred thoroughly with a magnetic bead to ensure a homogeneous blend. Subsequently, a 2N sodium hydroxide solution was added drop by drop until a pH 12 was achieved. The resulting solution was continuously stirred on a hot plate at 70 °C until the solution becomes dried. The obtained compound underwent calcination at 700 °C for 3 h in a muffle furnace.

### Synthesis of MgAl_2_O_4_ nanoparticle

MgAl_2_O_4_ nanoparticles were synthesized through a cost-effective sol–gel method. In this procedure, stoichiometric quantities of magnesium nitrate hexahydrate [Mg(NO_3_)_2_·6H_2_O], aluminium nitrate nonahydrate [Al(NO_3_)_3_·9H_2_O] and urea [NH_2_CONH_2_] were separately placed in a beaker. A 100 mL of deionized water was added to each, and the mixture was stirred thoroughly with a magnetic bead to achieve a homogeneous blend. A 2N sodium hydroxide solution was then added dropwise until a pH 12 was reached. The resulting solution underwent continuous stirring on a hot plate at 70 °C until the solution becomes dried. The obtained dried compound was subjected to calcination at 700 °C for 3 h in a muffle furnace.

### Synthesis of CaO:MgAl_2_O_4_ nanocomposite

**Fig. 1:**
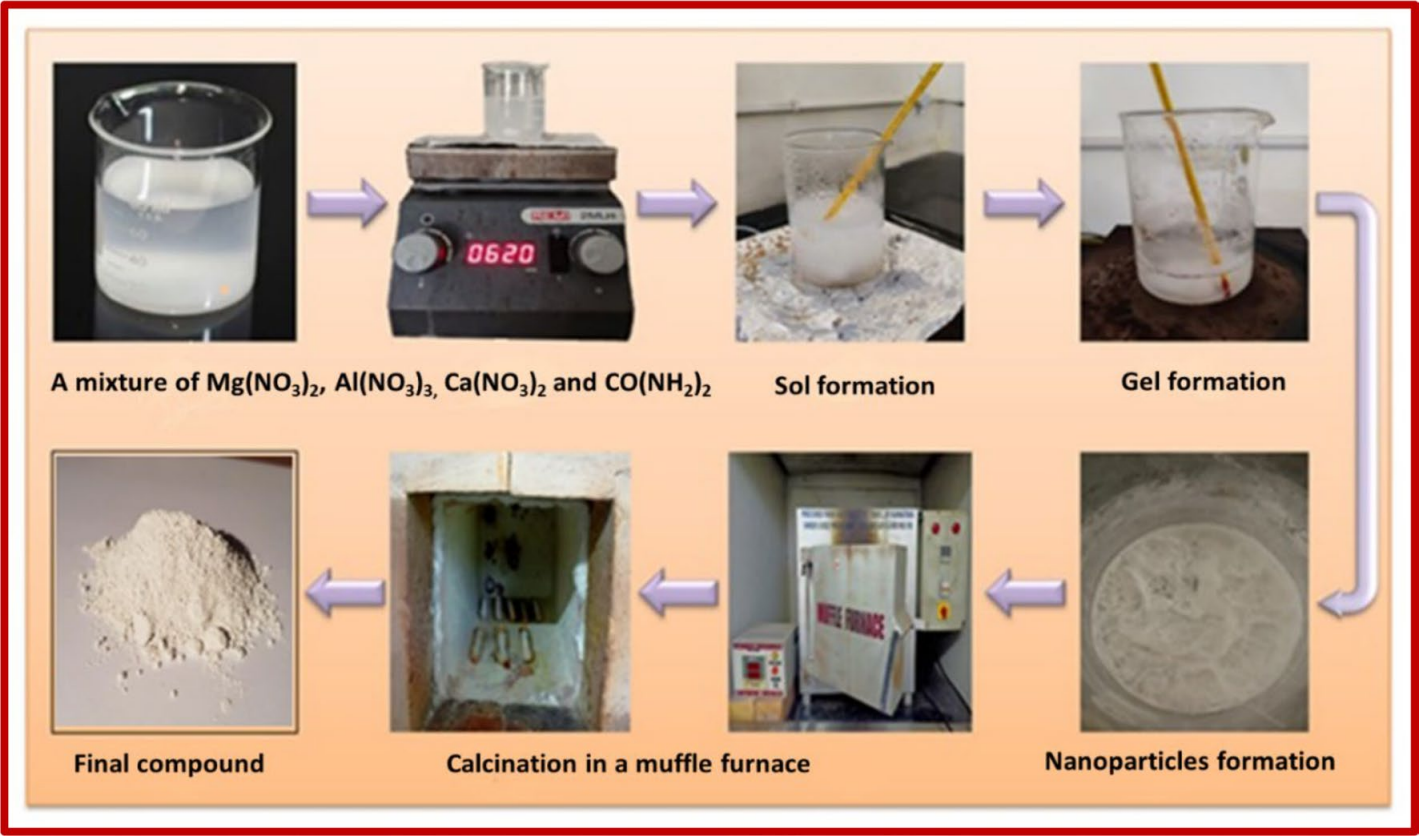
Schematic representation of synthesis of CaO:MgAl_2_O_4_ nanocomposite

A nanocomposite of CaO:MgAl_2_O_4_ in a 1:1 ratio was prepared using the sol–gel method with urea as a fuel. Starting materials included magnesium nitrate [Mg(NO_3_)_2_·6H_2_O], aluminium nitrate [Al(NO_3_)_3_·9H_2_O], calcium nitrate [Ca(NO_3_)_2_.4H_2_O], and urea [NH_2_CONH_2_]. The compounds were dissolved in a 100 mL of distilled water, and the stoichiometry of the redox mixture for sol–gel synthesis was calculated based on the total oxidizing and reducing valences of the compounds [23]. The mixture was stirred thoroughly to achieve a homogeneous blend. A 2 N Sodium hydroxide was added dropwise to achieve a pH 12, and the resulting solution was continuously stirred on a hot plate at 70 °C until the solution becomes dried. The obtained compound underwent calcination at 700 °C for 3 h in a muffle furnace. Figure 1 depicted the synthesis of the CaO:MgAl_2_O_4_ nanocomposite.

### Fabrication of modified carbon paste electrode

To create the modified carbon paste electrode, a mixture of the prepared CaO:MgAl_2_O_4_ nanocomposite, graphite powder, and silicone oil in a weight ratio of 15:70:15% was blended in an agate mortar for approximately 30 min. The resulting paste was loaded into a syringe tip attached to a copper wire and then evenly spread on paper until the surface achieved uniformity [24].

## Characterisations

The structural properties of the resulting CaO:MgAl_2_O_4_ nanocomposite were assessed utilizing monochromated Cu-Kα (1.5418 Å) radiation in a Shimadzu analytical X-ray diffractometer. DRS analyses in the 200–800 nm range were carried out using a Shimadzu UV–Vis 1800 double-beam spectrophotometer. The Perkin Elmer FTIR spectrometer was employed to record stretching frequencies and bending vibrations of the samples. Morphological analysis was performed using a TESCAN-VEGA3 LMU SEM instrument with EDXA technique. Transmission electron microscopy (TEM) analysis was performed on a JEOL JSM 2100 instrument. Cyclic voltammetry (CV) studies were conducted using a conventional three-electrode setup and a CHI608E potentiostat. Photocatalytic activities were assessed using Shimadzu’s UV–Visible spectrophotometer model 600.

### X-ray diffraction analysis

**Fig. 2:**
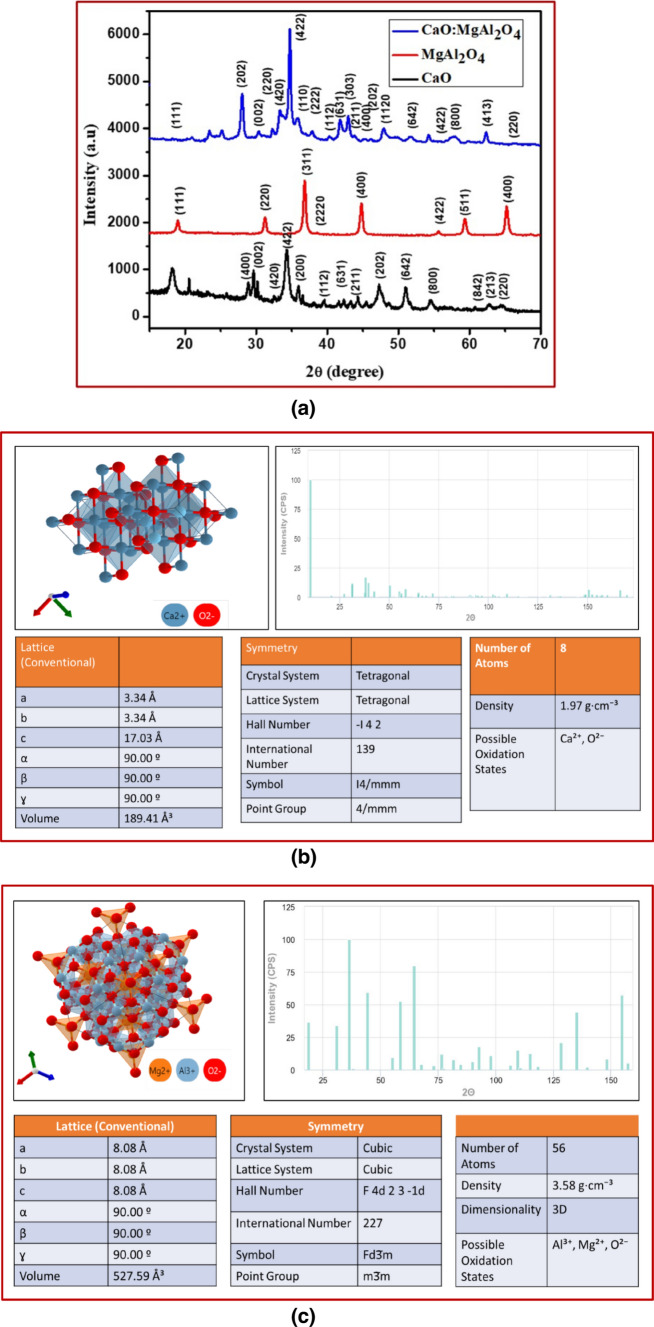
**a**. XRD patterns of CaO:MgAl_2_O_4_ nanocomposite, **b**. The X-ray diffraction patterns of CaO computed using a DFT framework sourced from a materials project, **c**. The X-ray diffraction patterns of MgAl_2_O_4_ computed using a DFT framework sourced from a materials project

The structural characterization of the prepared materials was carried out using the XRD technique. In Fig. 2a, XRD patterns for CaO, MgAl_2_O_4_, and the CaO:MgAl_2_O_4_ nanocomposite synthesized by the sol–gel method are presented. The diffraction peaks associated with crystal planes (400), (002), (420), (422), (200), (112), (211), (631), (211), (202), (642), (800), (842), (213) and (220) confirmed the tetragonal structure of CaO, consistent with JCPDS card number 00-01-085-0514. Similarly, the diffraction peaks corresponding to planes (111), (220), (311), (222), (400), (422), (511), (440), and (533) indicated the cubic structure of MgAl_2_O_4_, in good agreement with JCPDS card number 21-1152.

The CaO: MgAl_2_O_4_ (1:1) nanocomposite was confirmed to contain both CaO and MgAl_2_O_4_ components. The XRD pattern exhibited no additional peaks, indicating the absence of contaminants in the sample. The application of Scherrer’s formula allowed the determination of the average crystallite size (D) based on the broadening of the X-ray peaks.

1 $$D =\frac{K\lambda }{\beta cos\theta }$$

where, K is the constant, λ is the wavelength of the X-rays, β is the full width at half maximum of the intense peak in the XRD pattern and $\theta$ is the peak position [25].

The analysis revealed that the average crystallite size for CaO and MgAl_2_O_4_ nanoparticles was 28.83 nm and 20 nm, respectively. In the synthesized CaO:MgAl_2_O_4_ nanocomposite, the determined crystallite size was 24.15 nm. The peakes of CaO:MgAl_2_O_4_ nanocomposite at 2 $\uptheta$ values ranging from 18.23° to 65.03° are responsible to determine the crystalline size of the nanocomposite.

The Materials Project employs Density Functional Theory (DFT), an atomistic technique, for a comparative analysis of the structure of inorganic materials. This approach helps save time and resources in conducting structural characterizations [26]. The crystal structure of the oxide is built on a closely packed array of oxygen anions, with metal cations occupying interstitial sites. Typically, face-centered cubic and hexagonal close-packed arrays are utilized in these structures [27]. In this study, the lattice parameters were determined to be a = b = 3.288 Å, c = 6.72 Å, indicating a tetragonal structure, as illustrated in Fig. 2b.

The lattice parameters were determined to be a = b = c = 8.08 Å with cubic structure as shown in Fig. 2c.

### Fourier transform infrared (FTIR) spectroscopy analysis

**Fig. 3:**
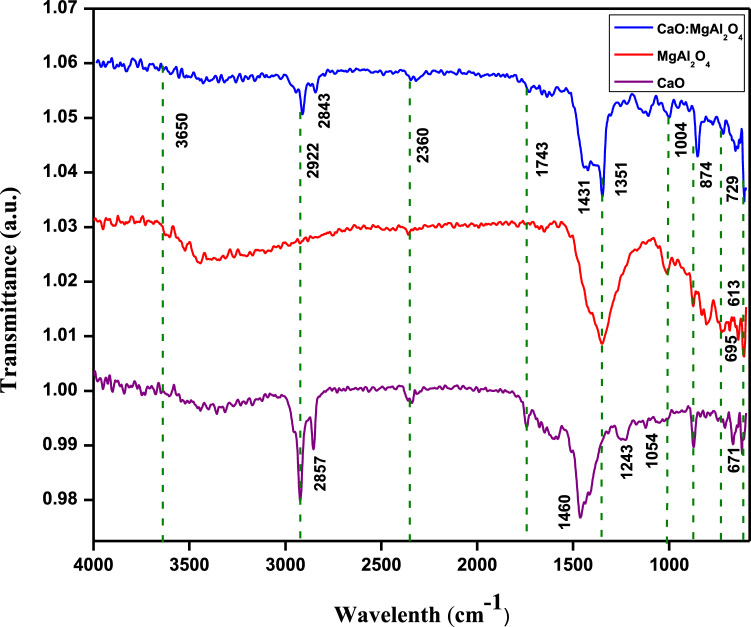
FT-IR analysis of CaO, MgAl_2_O_4_, CaO:MgAl_2_O_4_ nanocomposite

The structural formation of the CaO, MgAl_2_O_4_, and CaO:MgAl_2_O_4_ nanocomposites and the presence of functional groups on the surface were investigated through FT-IR spectral analysis. Figure 3 displays the FT-IR spectra of the synthesized materials, recorded within the wavenumber range of 4000 cm^−1^ to 500 cm^−1^. For CaO:MgAl_2_O_4_ nanocomposites, a high-intensity band at 1351 cm^−1^ was attributed to C–H bending vibrations, while the band at 1004 cm^−1^ corresponded to C–O stretching frequency. O–M–O bond bending vibrations were assigned to the frequency band at 874 cm^−1^ [28]. In the 400–800 cm^−1^ range, absorption peaks at 613 cm^−1^ and 695 cm^−1^ could be attributed to Mg–O stretching with lattice vibration and [AlO_6_] groups [29]. The peak at 2360 cm^−1^ resulted from metallic cations absorbing CO_2_. Peaks at 3650 cm^−1^ and 1743 cm^−1^, associated with O–H stretching vibration and H–O–H bending vibration, respectively, may be linked to adsorbed water molecules [30, 31]. The CaO:MgAl_2_O_4_ nanocomposite’s C–H stretching can be attributed to the band at 2857 cm^−1^, also 1460 cm^−1^, the O–H in-plane band are observed [32]. However, CaO nanoparticles had peaks at 1431 cm^−1^ and 1054 cm^−1^, which were attributed to the carbon–oxygen link and indicated that the particles were carbonated [33].

### Ultraviolet diffusion reflectance spectroscopy

**Fig. 4:**
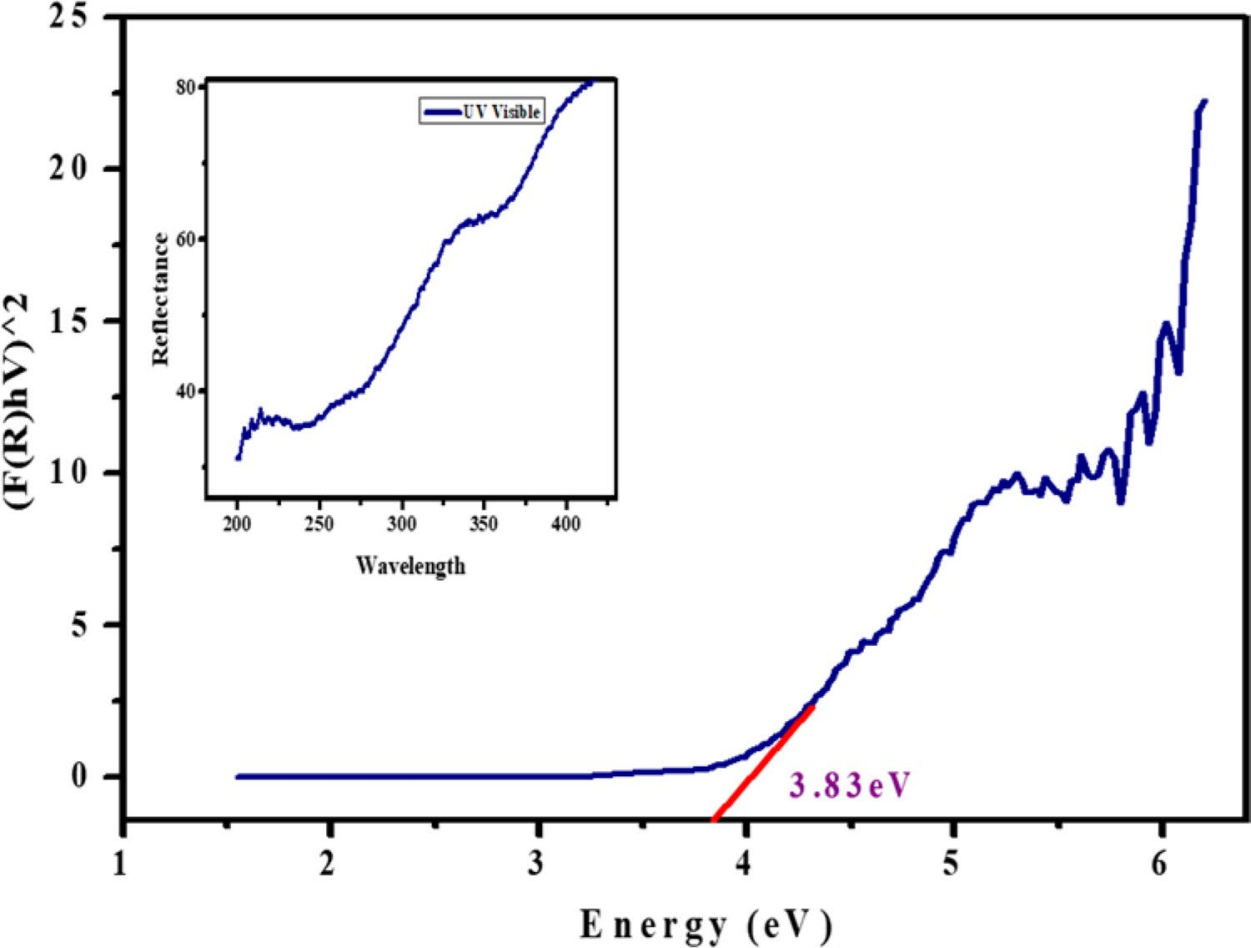
Energy band gap plot of CaO:MgAl_2_O_4_ nanocomposite

The Kubelka–Munk function, expressed by a specific equation, was employed to discern the nature of the band gap. This was achieved using a UV spectrophotometer in the diffuse reflectance mode to investigate the optical characteristics of the CaO:MgAl_2_O_4_ nanocomposite as shown in Fig. 4 [34].

2 $$F \left(R\right)= \frac{{\left(1- R\right)}^{2} }{2R}$$

where, R is the diffuse reflectance of the sample and F(R) is the Kulbeka-Munk function.

Tauc plot gives the relationship between the energy band gap and the absorption coefficient, which is expressed by equation,

3 $${\left[F\left(R\right)hv\right]}^{n }= A\left(h\upsilon - {E}_{g}\right)m$$

where, α is the linear absorption coefficient of the sample, hυ is the photon energy, A is an energy independent constant, Eg is the optical energy band gap and m is a constant depending on the band gap.

The direct energy band gap of the CaO:MgAl_2_O_4_ nanocomposite in a 1:1 ratio was determined to be 3.83 eV, as illustrated in Fig. 4. The synthesized composite exhibited reflectance wavelengths in the range of 200–375 nm, providing insights into the bandgap energy. The light absorption characteristics of the photocatalysts were significantly influenced by the CaO:MgAl_2_O_4_ nanocomposite.

### Scanning electron microscopy (SEM) and energy dispersive X-ray analysis

**Fig. 5:**
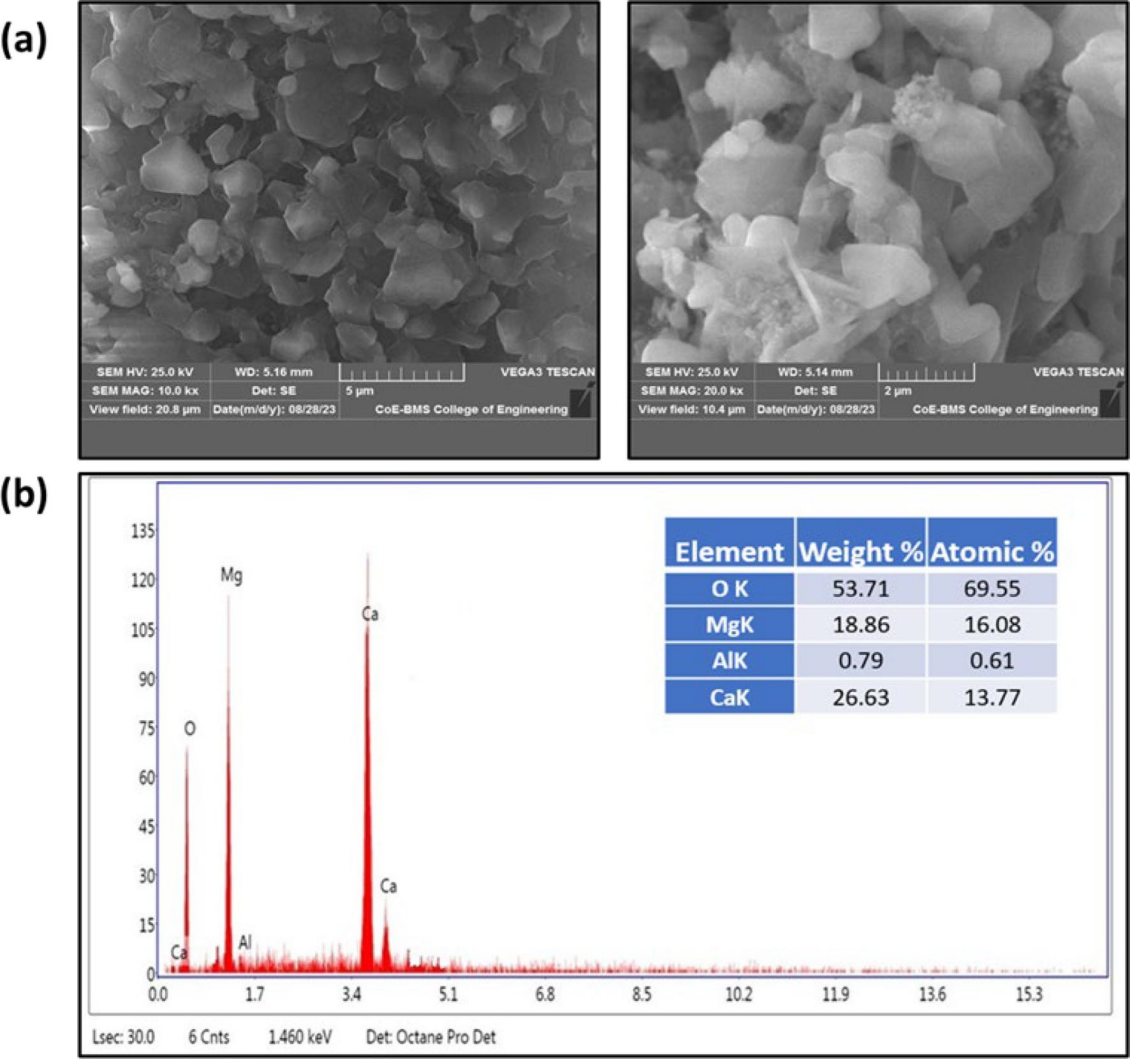
**a** SEM images at different magnification, **b** Electron dispersive X-ray of CaO:MgAl_2_O_4_ nanocomposite

Scanning electron microscopy (SEM) images were employed to scrutinize the surface properties of the synthesized nanocomposites [35]. In Fig. 5a, SEM pictures of the synthesized nanocomposites at a 1:1 ratio are presented. These images illustrate a uniform distribution of nanoparticles with a flake-like morphology influenced by the presence of urea.

Energy-dispersive X-ray (EDX) analysis spectra were utilized to determine the chemical composition and elemental content of the synthesized nanocomposites [35]. As depicted in Fig. 5b, specific peak patterns were observed, revealing the percentage composition of Ca, Al, Mg, and O ions in the EDX spectra. The percentage composition of the peaks correlates with their intensity at various energies. Notably, as the composite formed, it was observed that the peak intensities increased, indicating that the main components of the nanocomposite, such as calcium and magnesium, were present in appropriate amounts within the chemical composition [36, 37].

### TEM-HRTEM and SAED analysis

**Fig. 6:**
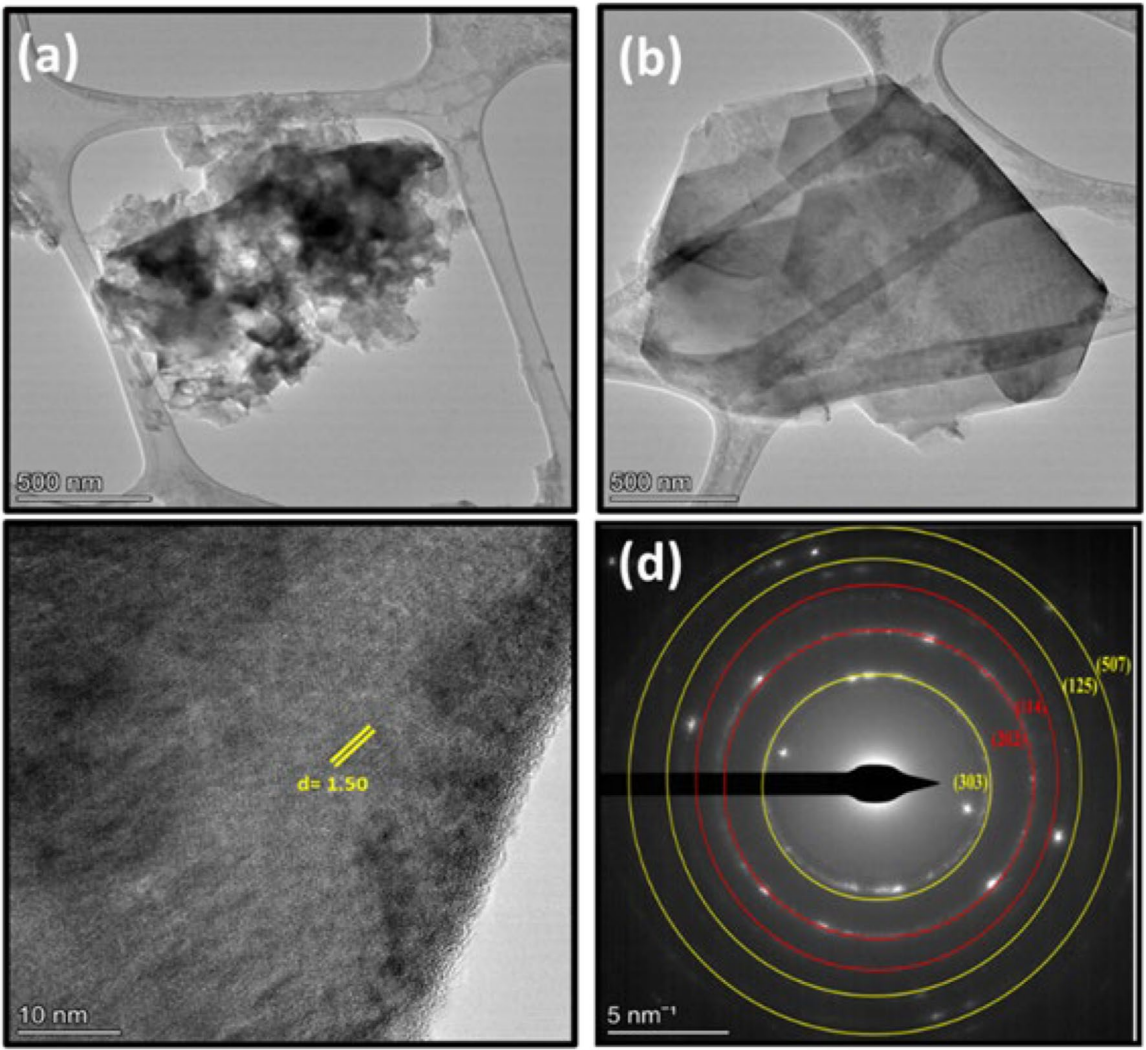
**a**–**b** TEM images, **c** HR-TEM and **d** SAED pattern of CaO:MgAl_2_O_4_ nanocomposite

Figure 6 shows the TEM, HR-TEM, and SAED pattern for the synthesized CaO:MgAl_2_O_4_ nanocomposite. The structure of the nanocomposite is evident in the TEM images. HRTEM image shows clear and distinct planes which indicates the nanocrystalline nature of the synthesized nanocomposite. Bright spots in the SAED pattern indicate the high crystalline nature of the material and the presence of planes are confirmed by the SAED pattern.

## Photocatalytic activity

Photocatalysis is a light-induced process facilitated by a catalyst. In this study, the photocatalytic activity of nanocomposites was investigated using AR-88 dye as an anionic dye model. A dye stock solution was prepared with distilled water to achieve the desired concentration. In a 250 ml circular glass reactor, 20 ppm of the AR-88 dye solution and 60 mg of the catalyst were added, and the reactor was then exposed to UV light radiation. UV–Vis measurements of dye solution concentration and absorbance were taken at the conclusion of the experiment [38].

**Fig. 7:**
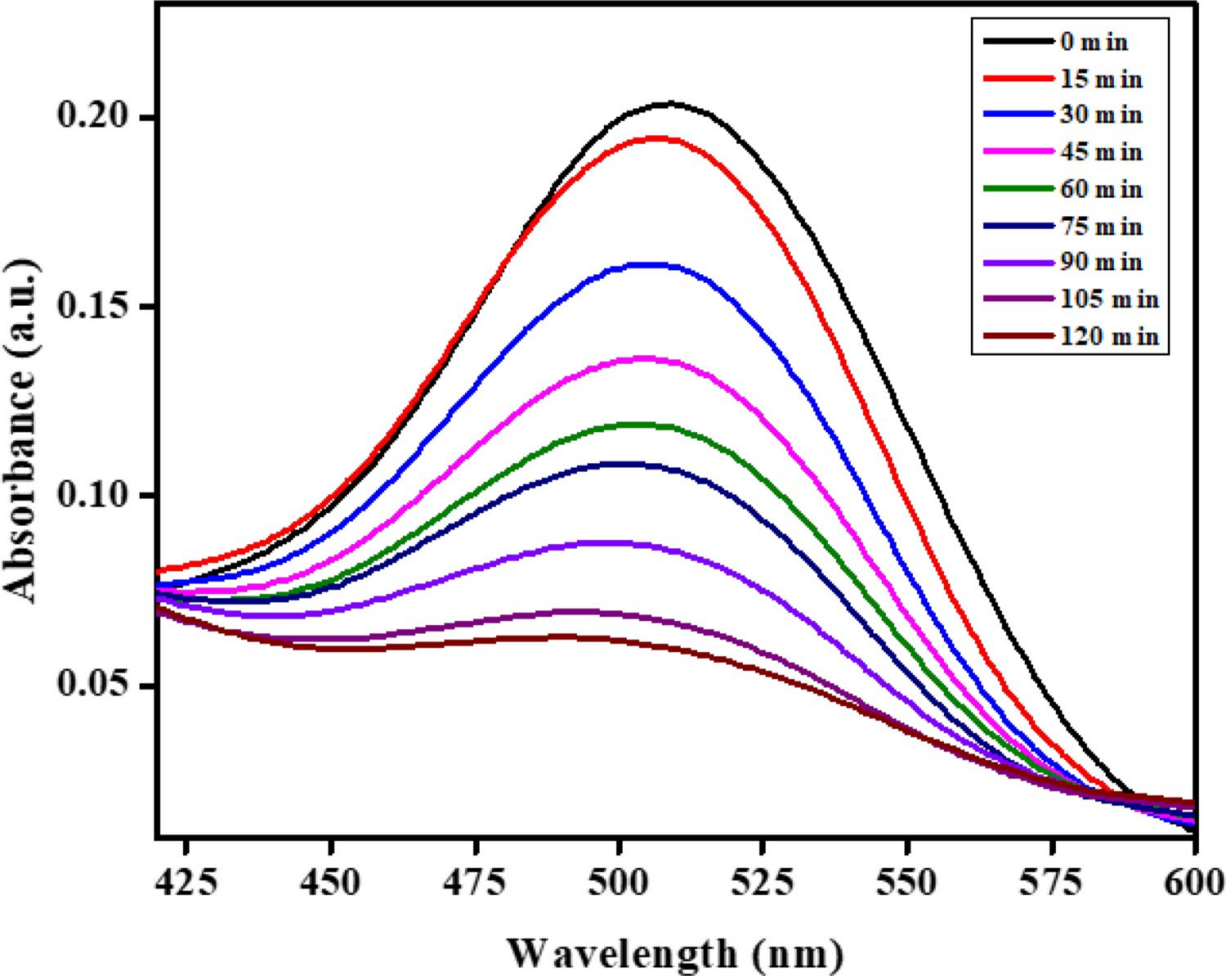
Photodegradation of AR-88 dye using CaO:MgAl_2_O_4_ nanocomposite as photocatalyst

As Fig. 7 shows, he maximum absorbance peak of AR-88 dye was identified at 509 nm. As the exposure time to ultraviolet light increased, the absorbance peaks gradually decreased in the presence of the catalyst, showcasing the photocatalytic capability of the synthesized nanocomposites. UV light, owing to its high energy, effectively supports photocatalysis [39]. With the material’s bandgap estimated to be approximately 3.83 eV based on previous discussions, the rapid degradation of AR-88 dye in the initial phase was attributed to the abundant availability of active sites on the catalyst.

**Fig. 8:**
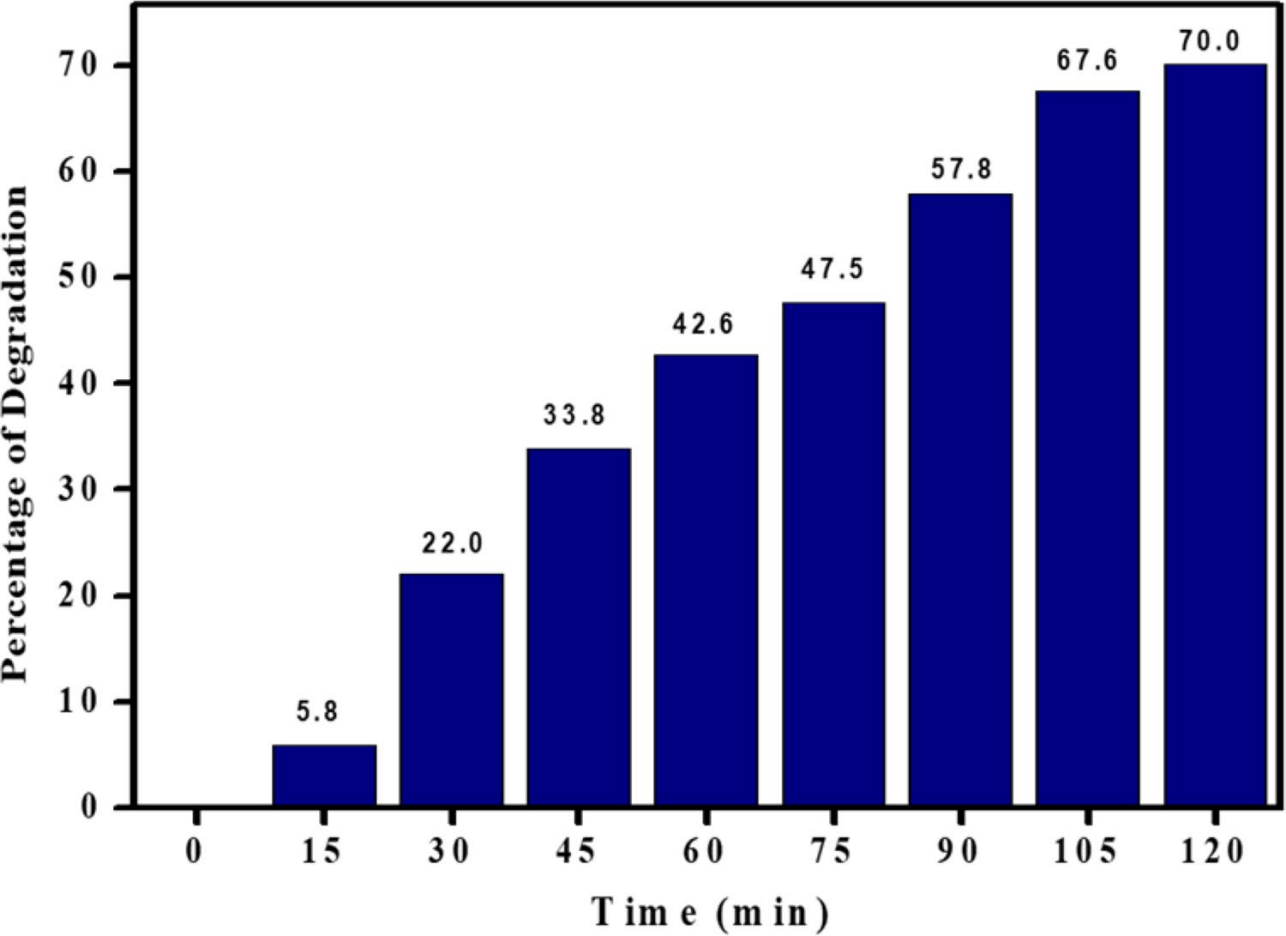
Variation of percentage degradation with respect to time of CaO:MgAl_2_O_4_ nanocomposite

Figure 8 illustrates the exposure of the synthesized composite to 120 min of UV light irradiation. Using the relevant formula and figure, the photodegradation efficiency of AR-88 dye was determined. Figure 7 exhibits a 70% degradation of the red acid dye, attributed to the uniform dispersion of the catalyst and a greater number of active sites on its surface. The degradation of the dye increased over time under UV light irradiation in the presence of the synthesized nanocomposite [40].

4 $$Percentage \,of\, degradation =\frac{{\text{C}}_{0 }-\text{ C}}{{\text{C}}_{0}} \times 100$$

where, C is the initial concentration of dye and C_0_ is the dye concentration at time t seconds after photodegradation.

**Fig. 9:**
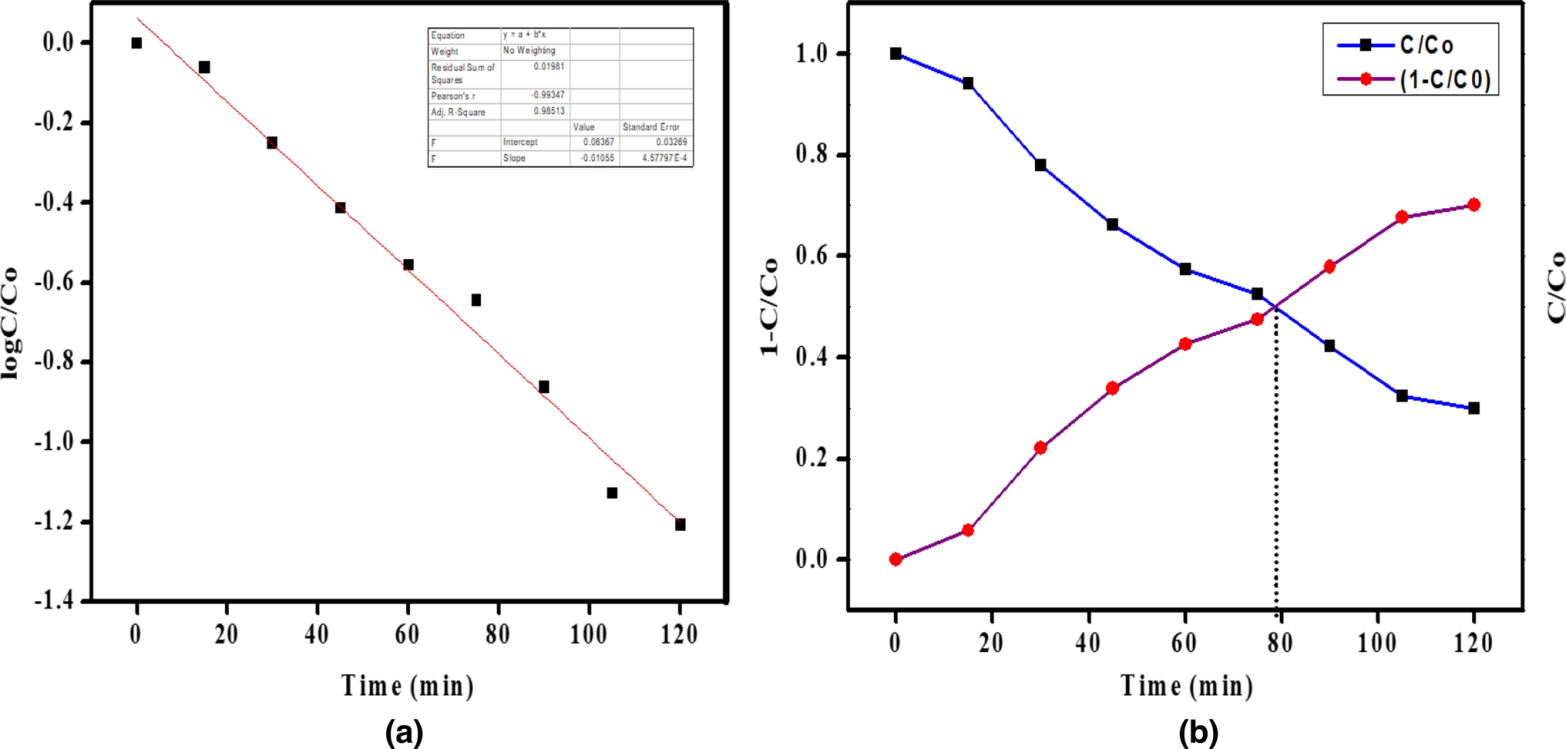
**a** Plot of C/C_0_ Vs time, **b** Half-life estimation for the degradation of AR-88 dye under UV light illumination using CaO:MgAl_2_O_4_ nanocomposite photocatalyst

The ratio C/Co for the decolorization of AR-88 dye under UV light irradiation is depicted in Fig. 9a, illustrating the improvement in degradation over time, reaching its peak at 120 min. The equation log(C/Co) was employed to evaluate the order of kinetics [41].

5 $$Log\frac{C}{{C}_{0}}= -kt$$

where, K is the first order rate constant, Co is the dye concentration at t = 0 min and C is the concentration of dye during testing.

The observed linearity between log(C/Co) and K indicates first-order kinetics. The value of K for red acid dye was determined to be 0.98513 min⁻^1^. Figure 9b presents the degradation half-life analysis, indicating 50% dye degradation in 79 min. These results suggest that the CaO:MgAl2O4 nanocomposite can be effectively utilized as a photocatalyst for the decolorization of AR-88 dye. This is attributed to its enhanced photodegradation capabilities, resulting from reduced electron–hole recombination and the generation of superoxide and hydroxyl radicals [42].

### Recyclability and reusability

**Fig. 10:**
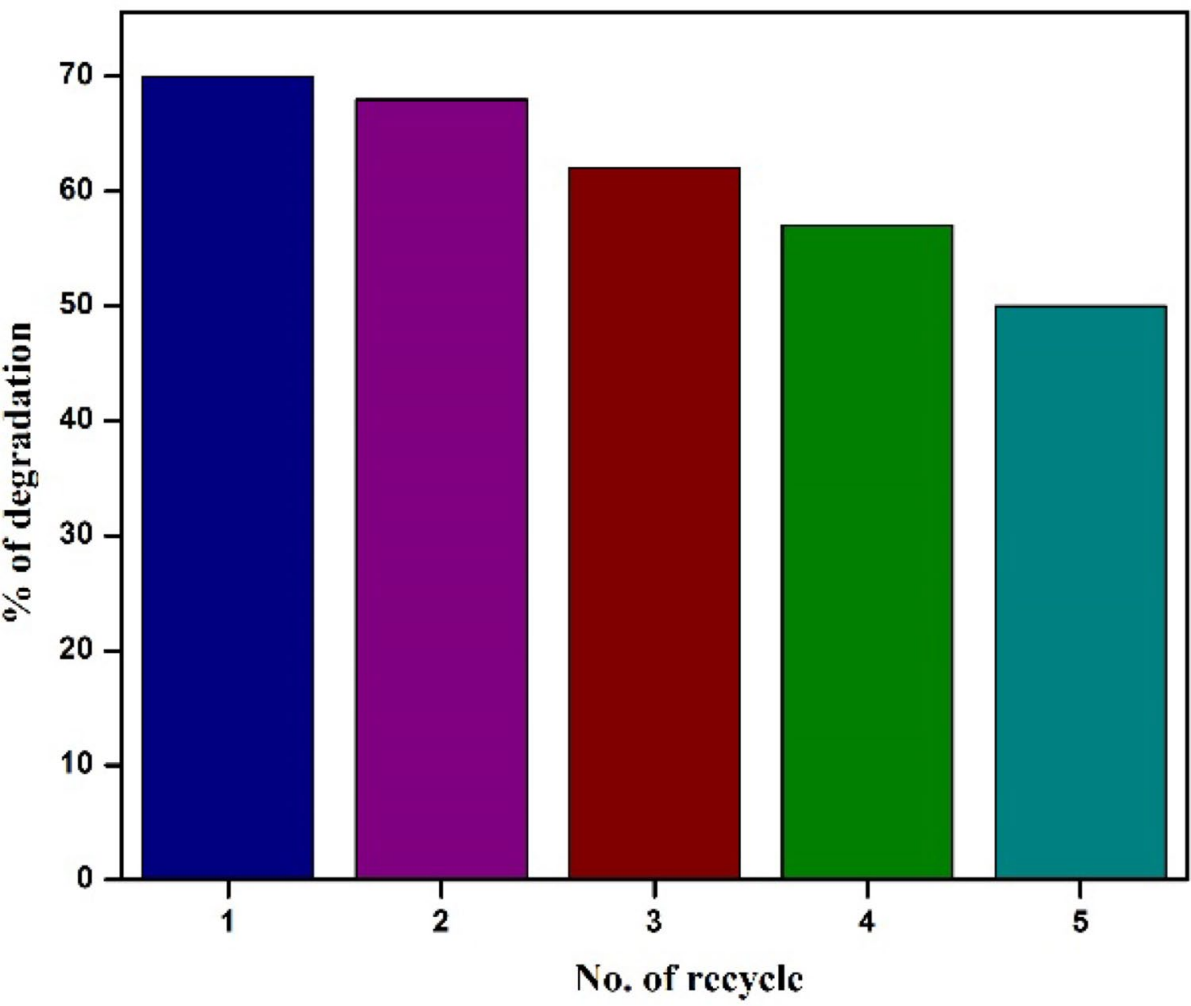
Reusability efficiency of AR-88 dye degradation using CaO:MgAl_2_O_4_ nanocomposite

The lifetime of the catalyst is one of the fundamental factors in choosing an efficient one. Because it is essential to significantly reduce the cost of treating organic dye effluents. As a result, the CaO:MgAl_2_O_4_ photocatalyst’s reusability was assessed in comparison to the decolorization of acid red-88 dye for about five times [43]. As demonstrated in Fig. 10, in the first cycle, UV absorption spectra were first obtained, and the CaO:MgAl_2_O_4_ nanocomposite was dried out in order to regain its reactivity. The nanocomposite was then used as a catalyst for the second cycle of AR-88 dye degradation, which was carried out five times, demonstrating the effectiveness and recyclable nature of the CaO:MgAl_2_O_4_ nanocomposite with no noticeable reduction in activity, indicating remarkable reusability. This was done to assess the catalysts’ suitability for recycling and use. Also, demonstrates that there are high activity levels of regenerated catalysts, even with possible partial loss following each cycle [44–47].

### Scavenger studies

**Fig. 11:**
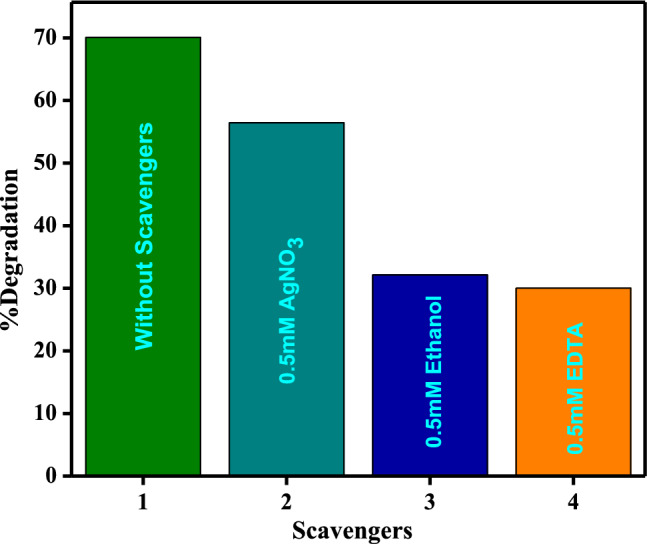
Effect of different scavengers on the photocatalytic degradation of AR- 88 dye solution using AgNO_3_, ethanol, EDTA

The scavenging study aims to identify the primary active species in photocatalysis, allowing for the evaluation of the mechanism of CaO:MgAl_2_O_4_ nanocomposite-catalyzed AR-88 degradation [48]. Degradation of the dye AR-88 was examined in the presence of 0.5 mM AgNO_3_, 0.5 mM ethanol, and 0.5 mM EDTA as scavengers. As depicted in Fig. 11, the degradation of the dye is more pronounced and intense in the absence of scavengers. In the presence of AgNO_3_, ethanol, and EDTA scavengers, the effectiveness of AR-88 dye degradation decreases. Scavenging tests confirmed that highly energetic free radicals effectively decolorize AR-88 dye when CaO:MgAl_2_O_4_ nanocomposite is used as a photocatalyst.

In this investigation, the photodegradation and analysis of three different scavengers, namely AgNO_3_, ethanol, and EDTA, were conducted. The percentages of degradation were determined to be 56.43%, 32.14%, and 30.03%, respectively. The inclusion of OH• and h^+^ scavenger suppresses the photocatalytic activity of CaO:MgAl_2_O_4_ nanocomposite. When the dye absorbs UV light, electrons are excited from the valence band to the conduction band, creating holes in the valence band. The catalyst in the sample’s conduction band then captures the electron. Water combines with hole-produced hydroxyl radicals to generate superoxide radicals when electrons and atmospheric oxygen combine [49].

In the photodegradation study, 70.03% degradation was achieved after 120 min without scavengers. AgNO_3_ blocks e-ions, ethanol blocks OH· radicals, and EDTA blocks h^+^ ions. The obtained results verified that the primary cause for the degradation of the dye compound is h^+^ ions [50].

**Fig. 12:**
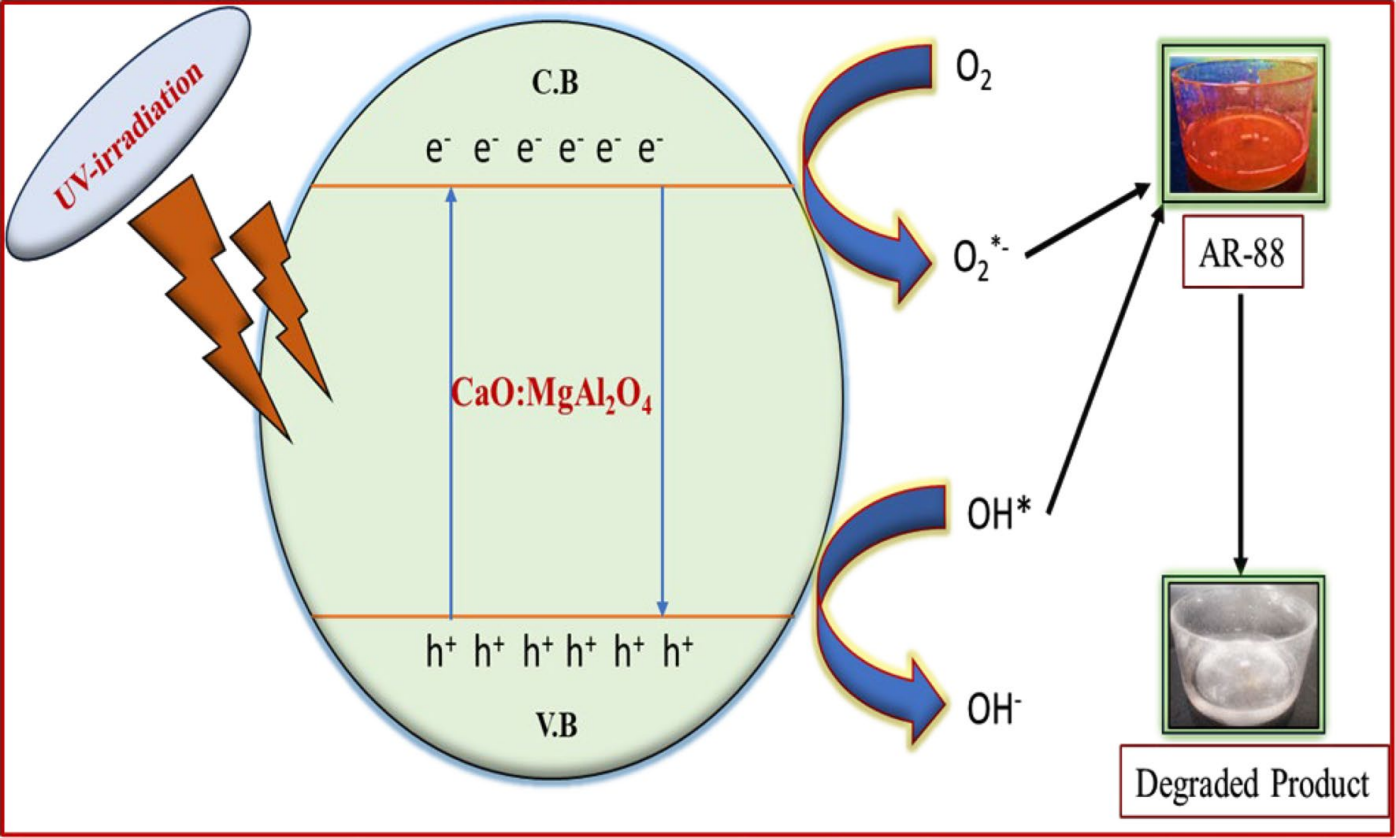
Possible mechanism for the degradation of AR-88 dye in presence of CaO:MgAl_2_O_4_ nanocomposite as photocatalyst

The photocatalytic degradation mechanism (Fig. 12) of organic dyes under ultraviolet (UV) radiation is expressed by the following equations,

$${\text{CaO}}:{\text{MgAl}}_{{2}} {\text{O}}_{{4}} {\text{nanocomposite}}\mathop{\longrightarrow}\limits^{{\text{UV light irradiation}}}{\text{CaO}}:{\text{MgAl}}_{{2}} {\text{O}}_{{4}} * \, \left( {{\text{Energy}}} \right)$$

$${\text{CaO}}:{\text{MgAl}}_{{2}} {\text{O}}_{{4}} * \, \left( {{\text{Energy}}} \right) \to {\text{h}}^{ + } + {\text{e}}^{ - }$$

$${\text{e}}^{ - } + {\text{O}}_{{{2} }} \to {\text{O}}_{2}^{ \cdot - } \left( {\text{Superoxide radical}} \right)$$

$${\text{2e}}^{ - } + {\text{O}}_{{{2}^{ - } }} + {\text{H}}_{{2}} {\text{O}} \to {\text{H}}_{{2}} {\text{O}}_{{2}}$$

$${\text{e}}^{ - } + {\text{H}}_{{2}} {\text{O}}_{{2}} \to {\text{OH}}^{ \cdot } + {\text{OH}}^{ - }$$

$${\text{dye}} + {\text{OH}}^{ \cdot } + {\text{O}}_{{2}} \to {\text{CO}}_{{2}} + {\text{H}}_{{2}} {\text{O}} + {\text{other degradation products}}$$

### Effects of wastewater treatment using synthesised nanocomposite on plant growth:

The assessment of treated wastewater toxicity is crucial for overcoming challenges in widespread wastewater treatment and reuse. In order to evaluate the appropriateness and sustainability of the photocatalytic process for potential wastewater reuse, a simple ecotoxicological bioassay involving the growth of plants from germinated seeds was conducted [51].

The photocatalytic process is a very efficient way to break down different kinds of dyes, and as we discussed in the previous section (dye degradation mechanism), it produces primarily harmless byproducts. Excellent growth activity is produced by the photocatalytic dye degradation byproducts, which serve as a source of fertiliser for the developing plants.

**Fig. 13:**
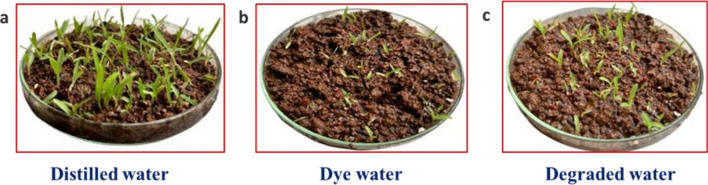
The growth of Eleusine coracana L. plants using **a** distilled water, **b** dye water, **c** dye degraded water

The growth of Eleusine coracana L. plants was studied concerning the effects of dyes both before and after photodegradation using synthesized CaO:MgAl_2_O_4_ nanocomposites. Eleusine coracana L. was chosen due to its delicate nature and rapid plant. As illustrated in Fig. 13, Eleusine coracana L. seedlings were cultivated in distilled water, polluted water containing dyes, and water treated with degraded dye solutions. As Fig. 13a shows, after five days of germination, the growth of Eleusine coracana L. seedlings in distilled water treated to promote plant growth was observed due to less toxicity in the distilled water. Whereas, in Fig. 13b, the plants in the AR-88 dye solution exhibited slow growth, and the presence of harmful chemicals caused a subsequent slowdown in plant activity. Also, the dye molecule destroys the seeds plasma membrane, which is explains the dye water sample shows no signs of plant development and it prevents sunlight penetration, which is quite harmful to photosynthesis [52].

However, the growth patterns of Eleusine coracana L. plants cultivated is more in photodegraded treated water and it is closely resembled those of the control plants (treated with distilled water) as showed in 13c. Because it contains least amount of toxic dye that does not impact the photosynthesis process as much as plant growth using dye water. Dye degradation reduces pollutants, improves water quality, and shields ecosystems and public health [53].

The study shows promising results for the safe use of treated wastewater. These results indicate that the treated wastewater can be safely utilized and it permits us to suppose that the synthesised nanocomposite acts as an excellent photocatalyst for the decolorization of AR-88 dye, and the degraded water sample may be used for agricultural purposes [54]. The concentration of dye solutions containing hazardous chemical components severely inhibited Eleusine coracana L. growth before degradation, highlighting the potential of the photocatalytic process for improving water quality and promoting safe plant growth. Higher atmospheric carbon dioxide concentrations, water molecules and sunlight are required to support the photosynthesis, which in turn promotes more plant growth. Also, partial degraded products, such as larger organic dye molecules, can be broken down into smaller fragments that are still organic but less complex are responsible for better plant growth in photodegraded water [55].

As seen in dye degradation mechanism, photodegradation is a partial degradation of dye caused by exposure to UV light on a CaO:MgAl2O4 nanocomposite material. This results in changes in the molecular structure of the dye molecules, including breaking chemical bonds within the dye molecules. As a result, the material loses its colour illumination over time as the dye molecules become less stable and cannot retain their original colour and intensity. These alterations in molecular structure often result in changes in colour, vibrancy, and stability of the dye, ultimately leading to partial degradation.

## Electrochemical analysis

### Cyclic voltammetry (CV)

**Fig. 14:**
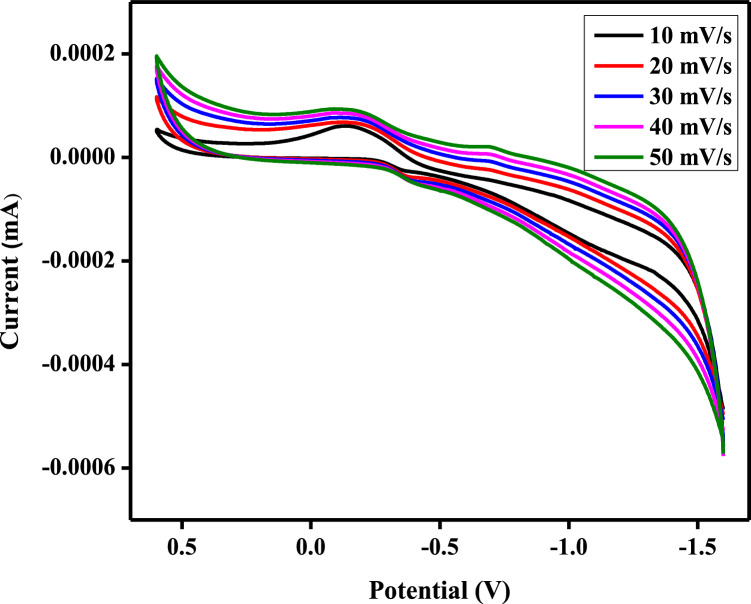
Cyclic voltammogram of CaO:MgAl_2_O_4_ nanocomposite

In a three-electrode system, which included a saturated Ag/AgCl electrode as a reference electrode, a platinum electrode as a counter electrode, and a modified composite carbon paste electrode as a working electrode, operating at room temperature, the cyclic voltammetry (CV) curves of the CaO:MgAl_2_O_4_ nanocomposite electrode were obtained in a 1 M KCl aqueous electrolyte solution. The CV curves were recorded at different scan rates (10, 20, 30, 40, and 50 mVs^−1^) with a potential range of 0.5 V to − 1.5 V, as shown in Fig. 12. The CV exhibited a small reduction peak at a potential of − 0.15 V, and it was asymmetric and nearly rectangular in shape. Figure 14 displayed a significant shift towards a positive potential, and a linear increase in the cathodic peak current was observed concerning the scan rate.

To determine the specific capacitance for the prepared electrodes based on the CV curves, the following formula can be employed [56].

6 $${C}_{sp}=\frac{S}{2mK\Delta V}$$

where, C_p_ is the specific capacitance, S is the area of the curve, m is the mass of the material, K is the scan rate of CV and V_1_–V_2_ is the potential window of CV.

At a sufficiently low scan rate of 10 mVs^−1^, the specific capacitance of prepared nanocomposite electrode was determined to be 125 Fg^−1^. Similarly specific capacitance for the 20 mVs^−1^, 30 mVs^−1^, 40 mVs^−1^, 50 mVs^−1^ scan rates was determined to be 64 Fg^−1^, 45 Fg^−1^, 39 Fg^−1^, 33 Fg^−1^ respectively as demonstrated in Fig. 12. At higher scan rates, the specific capacitance decreases because it becomes greater difficulty for the electrolyte ions to diffuse into the electrode’s internal structure and pores [57].

### Electrochemical impedance spectroscopy

**Fig. 15:**
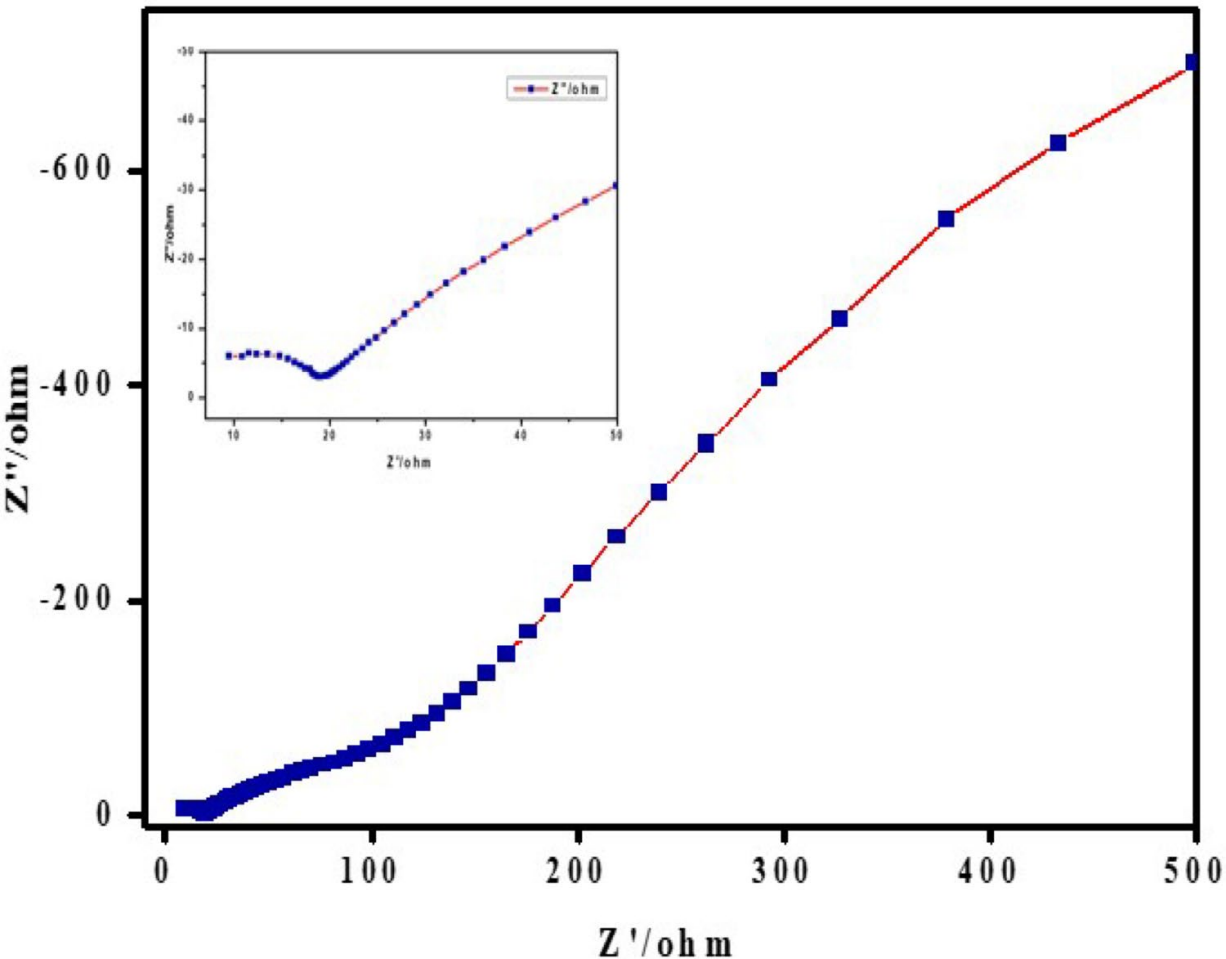
Nyquist plots for CaO:MgAl_2_O_4_ nanocomposite electrode in KCl

Electrochemical impedance spectroscopy (EIS) measurements were conducted to validate the observations made from cyclic voltammetry (CV) regarding the charge transfer capacity and conductivity of the CaO:MgAl_2_O_4_ nanocomposite’s prepared working electrode. Figure 15 illustrates the Nyquist plot of the prepared composite electrode. The semicircles in the Nyquist plots were utilized to determine the resistance at the active electrode and electrolyte interface. The arc radius represents the region of high frequency, while the line represents the region of low frequency.

The Nyquist plot indicates that the CaO:MgAl_2_O_4_ nanocomposite exhibited a reduced arc with a smaller diameter in the high-frequency region of the impedance spectrum in 1 M KCl, signifying high capacitance (C) and low charge transfer resistance (R_ct_) [58]. This indicating that the nanocomposite enhances the internal charge-transfer mechanism of the electrode. The study provides valuable insights into the internal resistance of the electrode material and the resistance between the electrode and the electrolyte [59].

### Electrochemical sensor for paracetamol detection:

**Fig. 16:**
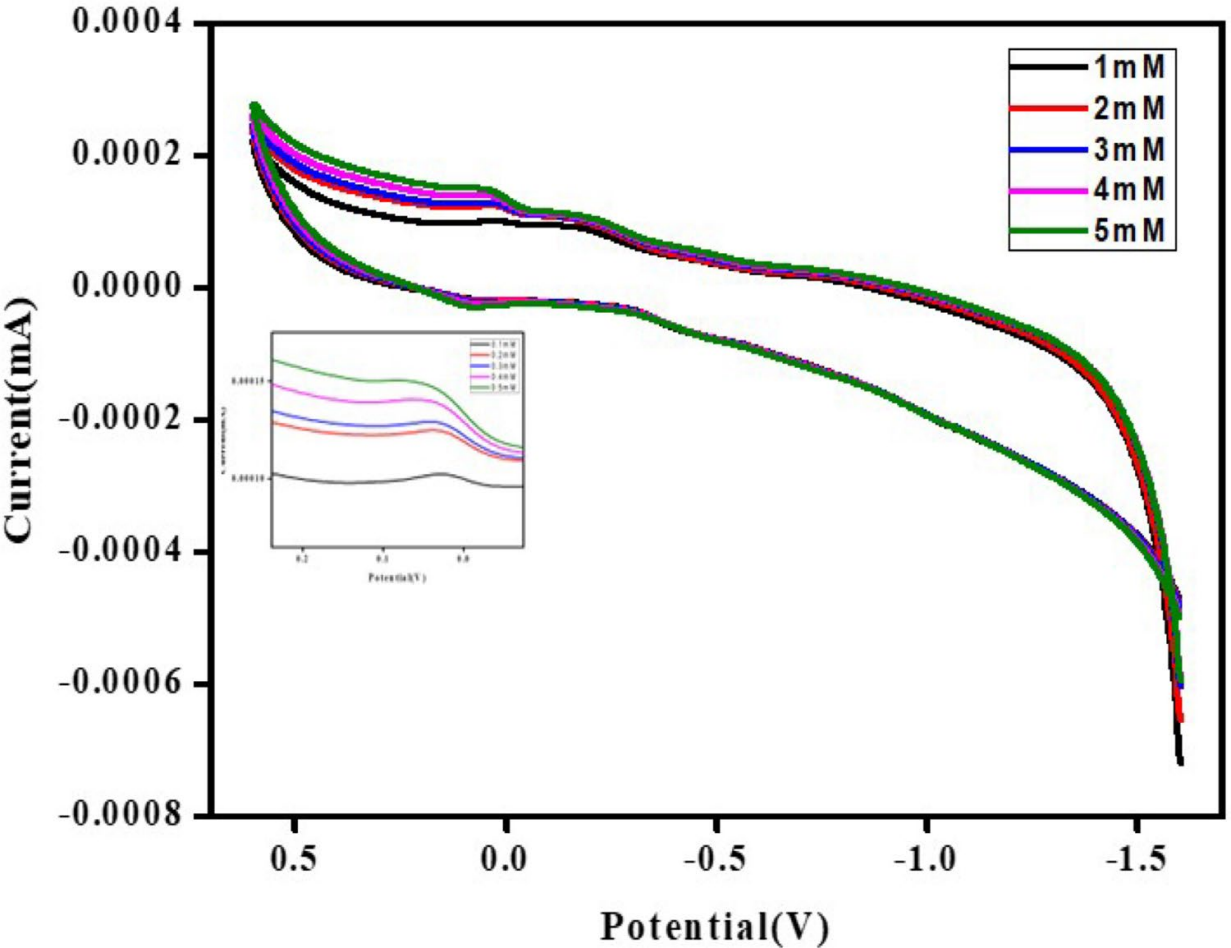
CV curves for paracetamol drug sensing using prepared CaO:MgAl_2_O_4_ nanocomposite electrode

The presence of paracetamol in 1 M KCl was detected using the cyclic voltammogram for the CaO:MgAl_2_O_4_ nanocomposite electrode. Paracetamol, commonly used for managing moderate to severe pain, was analyzed through electrochemical sensing, offering a method for determining substances in various mediums such as pharmaceutical formulations, body fluids, and water samples. Figure 16 illustrates the cyclic voltammetry (CV) analysis of the CaO:MgAl_2_O_4_ nanocomposite electrodes. A redox peak current was observed at 0.03 V, and it increased with the inclusion of paracetamol concentration, accompanied by a slight leftward shift in peak position. This shift may be attributed to the accumulation of an additive on the electrode’s surface.

### Electrochemical sensor for ascorbic acid detection

**Fig. 17:**
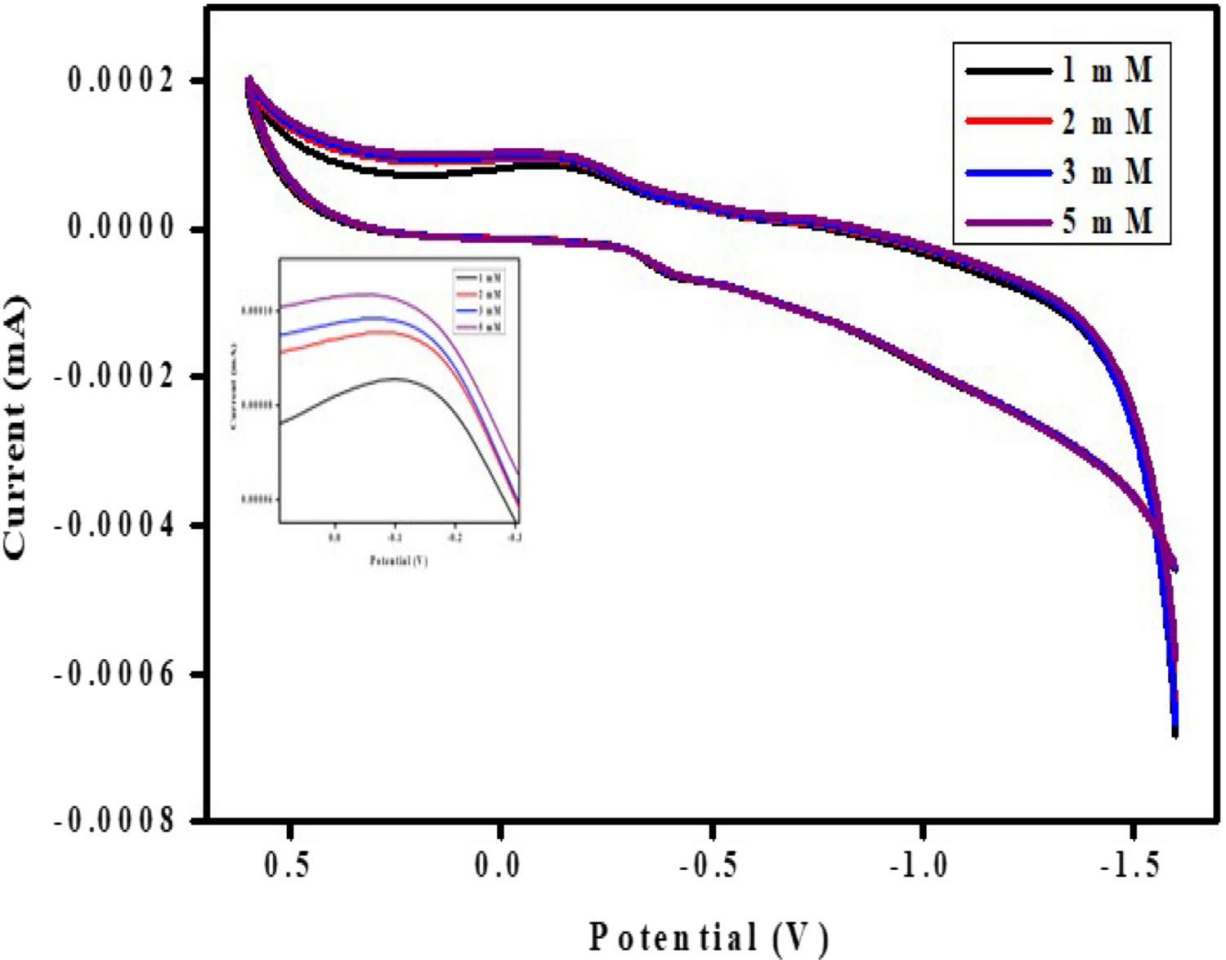
CV curves of prepared CaO:MgAl_2_O_4_ nanocomposite electrode for the detection of ascorbic acid

Figure 17 presents the cyclic voltammogram for the CaO:MgAl_2_O_4_ nanocomposite electrode used for the detection of ascorbic acid in 1 M KCl. A constant potential in the range from 0.6 to 1.6 V was applied to the prepared working electrode. The cyclic voltammetry (CV) for ascorbic acid detection in 1 M KCl exhibits a cathodic reduction peak at − 1.33 V. An increase in cathodic current was observed with increasing ascorbic acid content, and the cathodic peak position slightly shifted towards the left side [60].

Table 1 shows the comparative analysis of present work and literature. Compared to the literature, the present work exhibits superior applications. Further, it explores the path for further research to optimise energy and environmental applications.

**Table 1:** Comparative analysis of present work and literature

Material	Percentage dye degradation	Specific capacitance	Reference
CaO:MgAl_2_O_4_	70%	125 Fg^−1^	Present work
ZrO_2_:Tb^3+^	70%	–	61
Chitosan-Zinc Oxide hybrid composite	64%	–	62
NiCuF	–	16.9 Fg^−1^	63
NiCoF	–	60.0 Fg^−1^	63
RGO-PANI-c-ZnO	47%	–	64
PANI-BiVO_4_-GO	62%	–	65
Multi-walled CNT decorated V doped TiO_2_	65%	–	66
Wo_3_ on multiwalled CNT surface	65%	–	67
Graphene–CNT composites	–	110 Fg^−1^	68
MnO2 nanorods–rGO/V_2_O_5_ NWs–rGO	–	36.9 Fg^−1^	68
CoFe_2_O_4_/rGO nanocomposite	–	123.3 Fg^−1^	69
ZnO–CuO nanocomposite	49%	–	70
CuFe_2_O_4_/ZnO nanofibers	30.5%	–	71
TiO_2_/CuFe_2_O_4_	56%	–	72

In this present work, the CaO:MgAl_2_O_4_ photocatalyst demonstrated a 70% dye degradation of acid red-88 after 120 min under ultraviolet light conditions and exhibited a specific capacitance of 125 Fg^−1^. The RGO-PANI-c-ZnO nanocomposites, assisted by reduced graphene oxide and polyaniline, have been found to significantly improve the photocatalytic degradation of pharmaceutical antibiotics and methylene blue dye by 47% using natural sunlight [65]. The PANI-BiVO_4_-GO photocatalyst exhibited a 62% degradation of Rhodamine-B after 180 min under visible light conditions [66]. Ping He et al. fabricated supercapacitor electrodes using CoFe_2_O_4_/rGO nanocomposites and achieved a specific capacitance of 123.3 Fg^−1^ [69].The ZnO/CuO photocatalyst exhibited a 49% degradation of methylene blue after 300 min under visible light conditions [70].

## Conclusion

The synthesis of novel CaO:MgAl_2_O_4_ nanocomposites via the sol–gel method yielded promising results, with thorough characterization revealing structural, morphological, and electrochemical attributes. XRD studies affirmed an average crystalline size of 24.15 nm, while UV-DRS analysis estimated an optical band gap energy of 3.83 eV. Photocatalytic prowess was demonstrated through the effective degradation of AR-88 dye, achieving a notable 70% degradation within 120 min under UV irradiation. The CaO:MgAl2O4 nanocomposite electrode demonstrated excellent conductivity, reversibility, and biomolecule detection capabilities, particularly in identifying paracetamol and ascorbic acid during electrochemical sensing. Electrochemical evaluations in 1 M KCl demonstrated robust performance. Notably, the specific capacitance of the CaO:MgAl_2_O_4_ nanocomposite electrode exhibited dependence on scan rate 125 Fg^−1^, 64 Fg^−1^, 45 Fg^−1^, 39 Fg^−1^, and 33 Fg^−1^ for scan rates of 10 mVs^−1^, 20 mVs^−1^, 30 mVs^−1^, 40 mVs^−1^, and 50 mVs^−1^, respectively. The specific capacitance observed during paracetamol drug and ascorbic acid sensing underscored the electrode’s potential for the development of an efficient electrochemical sensor. Overall, the research presents a comprehensive exploration of the CaO:MgAl_2_O_4_ nanocomposite’s multifaceted properties, showcasing its potential across various applications.

## Data Availability

Data sets generated during the current study are available from the corresponding author on reasonable request.
